# Construction of a novel anoikis-related prognostic model and analysis of its correlation with infiltration of immune cells in neuroblastoma

**DOI:** 10.3389/fimmu.2023.1135617

**Published:** 2023-04-04

**Authors:** Ji Chen, Mengjiao Sun, Chuqin Chen, Meiyun Kang, Bo Qian, Jing Sun, Xiaopeng Ma, Jianfeng Zhou, Lei Huang, Bin Jiang, Yongjun Fang

**Affiliations:** ^1^ Department of General Surgery, Children’s Hospital of Nanjing Medical University, Nanjing, China; ^2^ Department of Hematology and Oncology, Children’s Hospital of Nanjing Medical University, Nanjing, China; ^3^ Department of Cardiothoracic Surgery, Children’s Hospital of Nanjing Medical University, Nanjing, China

**Keywords:** neuroblastoma, anoikis, tumor microenvironment, immune cell, WGCNA, drug sensitivity

## Abstract

**Background:**

Anoikis resistance (AR) plays an important role in the process of metastasis, which is an important factor affecting the risk stage of neuroblastoma (NB). This study aims to construct an anoikis-related prognostic model and analyze the characteristics of hub genes, important pathways and tumor microenvironment of anoikis-related subtypes of NB, so as to provide help for the clinical diagnosis, treatment and research of NB.

**Methods:**

We combined transcriptome data of GSE49710 and E-MTAB-8248, screened anoikis-related genes (Args) closely related to the prognosis of NB by univariate cox regression analysis, and divided the samples into anoikis-related subtypes by consistent cluster analysis. WGCNA was used to screen hub genes, GSVA and GSEA were used to analyze the differentially enriched pathways between anoikis-related subtypes. We analyzed the infiltration levels of immune cells between different groups by SsGSEA and CIBERSORT. Lasso and multivariate regression analyses were used to construct a prognostic model. Finally, we analyzed drug sensitivity through the GDSC database.

**Results:**

721 cases and 283 Args were included in this study. All samples were grouped into two subtypes with different prognoses. The analyses of WGCNA, GSVA and GSEA suggested the existence of differentially expressed hub genes and important pathways in the two subtypes. We further constructed an anoikis-related prognostic model, in which 15 Args participated. This model had more advantages in evaluating the prognoses of NB than other commonly used clinical indicators. The infiltration levels of 9 immune cells were significantly different between different risk groups, and 13 Args involved in the model construction were correlated with the infiltration levels of immune cells. There was a relationship between the infiltration levels of 6 immune cells and riskscores. Finally, we screened 15 drugs with more obvious effects on NB in high-risk group.

**Conclusion:**

There are two anoikis-related subtypes with different prognoses in the population of NB. The anoikis-related prognostic model constructed in this study can accurately predict the prognoses of children with NB, and has a good guiding significance for clinical diagnosis, treatment and research of NB.

## Introduction

NB is a kind of malignant solid tumor originating from neural crest stem cells, which is highly heterogeneous and most prone to occur outside the central nervous system in children (1). Surgery combined with chemotherapy can achieve a good prognosis for low-risk (2) and intermediate-risk (3) NB, while the prognoses of patients with high-risk NB are still poor even with a combination of surgery (4, 5), chemotherapy (6–8), radiotherapy (9), bone marrow transplantation (10) and other treatments. As an important therapeutic method for malignant tumors, immunotherapy has been widely used in the treatment of relapsed and refractory NB. With the application of immunotherapy methods such as GD2 monoclonal antibody (11–13) and CAR-T (14–16), the prognoses of children with relapsed and refractory NB have been significantly improved. However, how to improve the prognosis of high-risk NB with metastasis is still a problem that needs to be solved. Previous studies have shown that the prognosis of NB with bone metastasis or bone marrow metastasis is generally poor (17). Therefore, exploring the mechanism of NB metastasis will help us better understand the biological characteristics of NB, and provide the basis for molecular targeted therapy of high-risk NB.

Metastasis is a very complex biological process. In general, cells attach to the extracellular matrix (ECM) through signal transduction and carry out cell division and differentiation in specific areas (18, 19). Apoptosis occurs when cells leave the ECM, a process known as anoikis (20). Subsequently, tumor cells can invade the vasculature and colonize in distant organs in the absence of ECM and intercellular signaling. This process is known as AR. Anoikis was first found in epithelial cells and endothelial cells, and then AR was found to be an important pathway for tumor metastasis (21–24). A large number of studies have shown that the tumor microenvironment (TME) is closely related to the metastasis of malignant tumors (25, 26), and immune cells play important roles in the TME (27–30). Therefore, we hypothesize that there might be some relationships between AR and the changes of TME.

**Figure 1:**
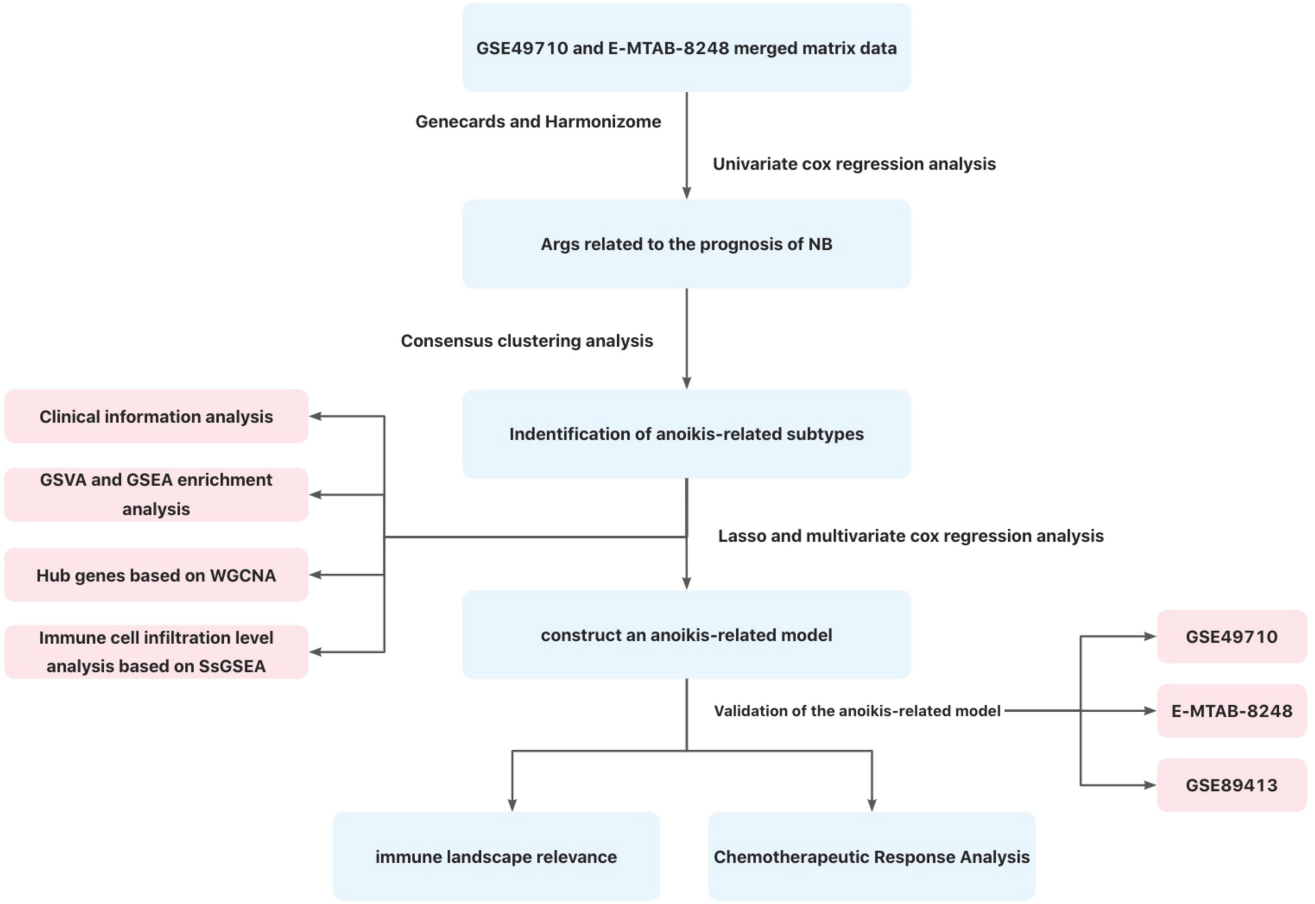
Flow chart of this study.

At present, the specific mechanism of anoikis in NB has not been elucidated (31, 32), and the relationship between AR and immune cell infiltration in NB is not clear. In this study, two anoikis-related subtypes of NB were screened, and the mechanisms of anoikis in NB were investigated by analyzing hub genes, important pathways and immune cell infiltration of anoikis-related subtypes based on transcriptome data from GEO and ArrayExpress databases. Subsequently, we constructed a reliable anoikis-related prognostic model of NB, and confirmed that this model had a good guiding significance in the evaluation of NB prognosis and the prediction of drug sensitivity. **Figure 1** illustrates the workflow of this research.

## Methods

### Data acquisition

Transcriptome data from GSE49710 (https://www.ncbi.nlm.nih.gov/geo/query/acc.cgi?acc=GSE49710) and E-MTAB-8248 cohort (https://www.ebi.ac.uk/arrayexpress/experiments/E-MTAB-8248/) in GEO and ArrayExpress databases was included in this study. The two cohorts included 498 and 223 patients respectively, and the main clinical information included age, sex, COG risk, *MYCN* status, INSS status, survival time, and survival status. The transcriptome data of 39 NB cell lines in GSE89413 cohort (https://www.ncbi.nlm.nih.gov/geo/query/acc.cgi?acc=GSE89413) was also obtained. After data correction and probe annotation, we finally got the transcriptome matrix data with row names of gene and column names of patient number/cell number. In order to obtain more accurate anoikis-related subtypes and build an anoikis-related prognostic model, we merged the transcriptome matrix data of GSE49710 and E-MTAB-8248, standardized them through the “normalizeBetweenArray ()” function, and finally got the merged matrix data.

### Args related to the prognosis of NB

Args were obtained by searching key word “anoikis” in Genecards (https://www.genecards.org/, score>0.4) and Harmonizome (https://maayanlab.cloud/Harmonizome/) databases. Through univariate cox regression analysis of the combined cohort, we screened Args closely related to survival status of children with NB by setting the standard of “HR≠1 and p<0.05”.

### Consensus clustering analysis for Args

We conducted consensus clustering analysis in 721 children by analyzing the expression levels of Args closely related to the survival status of NB. We finally obtained the best k-value for clustering by using the “CONSENSUSClusterPlus ()” function and setting the parameters reps=50, pItem=0.8, pFeature=1, clusterAlg=“km”, distance=“euclidean”, seed=123, and used the “prcomp ()” function to perform PCA analysis on all samples. Kaplan-Meier (KM) survival analysis of the anoikis-related subtypes was conducted by the “survdiff ()” function, and Args (|LogFC|>0.5 and p<0.05) differentially expressed in anoikis-related subtypes were screened through the “Lima” package. “Ggboxplot () “ and “ Pheeatmap () “ function were used to draw the boxplot and heatmap of differently expressed Args between different subtypes, and “ Pheeatmap () “ function was also used to observe the distribution of clinical indicators such as age, sex, COG risk, *MYCN* status and INSS stage between different subtypes.

### GSVA and GSEA enrichment analyses

We obtained the hallmark gene sets (c5.go.v7.4.symbols.gmt and c2.cp.Kegg.V7.4.Symbols.gmt) from MSigDB database, conducted GSVA and GSEA enrichment analyses between different subtypes through “GSVA” R package and “GSEA ()” function, and visualize the data through “pheatmap ()” and “gseaplot ()” function respectively.

### Analysis of TME

Single-sample gene set enrichment analysis (ssGSEA) was used to analyze the differences of immune cell infiltration between different anoikis-related subtypes. We firstly scored the infiltration levels of 28 kinds of immune cells in each NB sample by using the “GSVA ()” function, then analyzed the differences of immune cell infiltration in different subtypes, and finally visualized the data by using the “ggboxplot ()” function. We used “CIBERSORT ()” function to score immune cell infiltration level for each sample in different risk groups, analyzed the differences in immune cell infiltration level between different risk groups divided according to the prognostic model, and visualized the data by “vioplot ()” function. Finally, we used the “corrplot ()” function to visualize the correlation between immune cells. The “Cor.test()” function was used to analyze the correlation between immune cells and riskscores.

### Weighted correlation network analysis

By setting the standard of Height=2000, we used the “hclust ()” function to cluster all the samples and draw a clustering tree after filtering out the outliers. Based on the criterion of approximate scale-free network (R^2^>0.85), we used the “pick soft threshold ()” function to select an appropriate soft threshold to make the network more in line with the power law distribution, thus reducing the error and making the results more characteristic of biological data. We set 50 as the minimum number of genes in each module and 0.25 as the threshold of cutting height, used the dynamic cutting tree method to identify modules, combined the modules with high similarity and finally obtained multiple important modules. In order to get key modules, we evaluated the correlation between genes and samples by calculating gene significance (GS), defined module significance as the average GS of all genes in the module, calculated the correlation between the modules and anoikis, and selected the module with the highest correlation as the key module. By calculating the GS and module membership (MM) of each gene and setting the interception criteria of GS>0.5 and |MM|>0.80, we finally got the core genes of the key module. By overlapping the prognosis-related Args between two subtypes and the core genes screened by WGCNA, we finally screened the hub genes closely related to anoikis in NB.

### Construction and validation of anoikis-related prognostic model

All samples were randomly divided into training group and test group with equal sample size. Lasso regression analysis was performed on all Args related to the survival state in the training group through the “Glmnet ()” function. The minimum regularization parameter lambda (λ) and genes highly associated with anoikis-related subtypes were obtained by cross-validation (alpha=1). Multivariate cox regression analysis was then used to screen the core genes and the weight of each core gene through the “coxph ()” function. Riskscore of each sample was obtained according to the expression level and corresponding weight of core genes. The total sample group, training group and test group were divided into high-risk group and low-risk group according to the median riskscore value respectively. KM survival analysis was performed for the three groups by “survdiff ()” function. “pROC ()” function was used to calculate area under the curve (AUC) in three groups and evaluated the predictive capacity of this model. We used the “coxp ()” function to conduct multivariate cox regression analysis on the clinical information that might affect the prognoses of children with NB, and drew the nomogram through the “replot ()” function. The “calibrate ()” function and “plot ()” function were used to analyze the parameters of calibration curve and plot calibration curve respectively. The “survfit ()” function and “ggsurvplot ()” function were used to analyze the parameters of the cumulative hazard curve and plot the cumulative hazard curve respectively. The parameters of the decision curve were analyzed by “dca ()” function, and the decision curve was plotted by “ggplot ()” function.

### Drug sensitivity analysis

We obtained the training set gene expression matrix file (GDSC2_Expr.rds) and drug processing information file (GDSC2_Res.rds) from Genomics of Drug Sensitivity in Cancer (GDSC) database (https://www.cancerrxgene.org/). The “calcPhenotype ()” function in the “oncoPredict” R package was adopted, and the criteria of removeLowVaryingGenes = 0.2 and minNumSamples = 10 were set for the prediction of drug susceptibility.

### Data analysis

All data analyses in this study were completed by R 4.2.1 version, and p < 0.05 was considered statistically significant. The t test and one-way ANOVA were used to analyze the parametric data. The Wilcoxon test and Kruskal-Wallis test were used to analyze the nonparametric data of two and multiple independent samples respectively. Related R packages described above were downloaded from Bioconductor packages or R packages.

## Results

### Data acquisition and processing

In order to obtain relatively complete clinical data and transcriptome data with a large sample size, we selected GSE49710 and E-MTAB-8248 cohorts from GEO and ArrayExpress databases. The two cohorts included 498 and 223 clinical samples respectively. Both groups contained complete transcriptome array data and Log2 conversion was performed. The GSE49710 cohort contained relatively complete information of age (> 18 months n=193, ≤18 months n=305), sex (boy n=287, girl n=211), COG risk (High risk n=176, Low risk n=322), *MYCN* status (amplified n=92, non amplified n=401), INSS stage (Stage I n =121, Stage II n=78, Stage III n=63, Stage IV n=183, Stage IVs n=53), survival state (alive n=393, dead n=105) and survival time. E-MTAB-8248 had complete information of age (> 18 months n=119, ≤18 months n=104), *MYCN* status (amplified n=46, non amplified n=177), INSS stage (Stage I n =29, Stage II n=39, Stage III n=36, Stage IV n=89, Stage IVs n=30), alive state (alive n=181, dead n=42) and survival time. By combining and standardizing transcriptome data in two cohorts, we obtained transcriptome microarray matrix data with 721 samples. The transcriptome data of 39 NB cell lines in the GSE89413 cohort were obtained and Log2 conversion was also performed for further study.

### Consensus clustering analysis for Args

**Figure 2:**
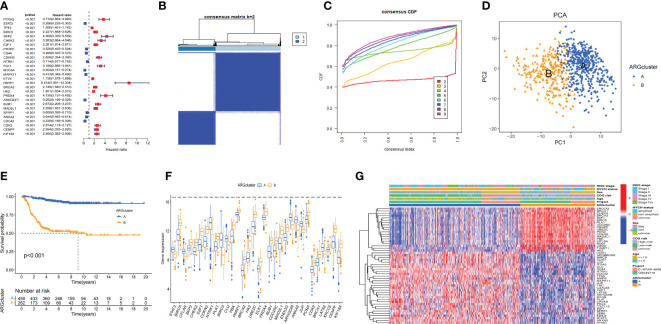
Consensus clustering analysis for Args **(A)**. Args closely related to the survival state were obtained according to the standard of HR≠1 and P<0.05. 28 genes with the lowest p-value through the forest map. **(B, C)**. Clustering heatmap and consistent cumulative distribution function (CDF) graph of 721 samples (k=2). **(D)**. Distinguish the samples into two subtypes through principal component analysis (PCA). **(E)** KM survival analysis of two anoikis-related subtypes. **(F)** Boxplot of 32 Args between the two subtypes with the lowest p-value. **(G)** Heatmap of clinical information and differentially expressed Args between ARCluster A and ARCluster B. ***, p < 0.001.

We searched the key word “anoikis” in Genecards and Harmonizome databases, and a total of 640 Args were obtained. Univariate cox regression analysis was conducted on the expression level of 640 genes and the survival state of the children in this study. A total of 283 Args closely related to the survival state were obtained according to the standard of HR≠1 and p<0.05. Ten Args with the greatest impact on the prognosis were: *PARP1* (HR=8.51), *ELAVL1* (HR=6.49), *XRCC5* (HR=5.62), *CSNK2A1* (HR=5.60), *DAP3* (HR=4.96), *MYO5A* (HR=0.20), *CDC42* (HR=0.24), *ARHGEF7* (HR=0.25), *PDPK1* (HR=0.27), *ATF2* (HR=0.27). Ten Args with the most statistical difference were: *NTRK1* (p=1.58e-35), *ETV4* (p=2.80e-31), *PARP1* (p=4.19e-30), *CDC42* (p=7.44e-30), *SKP2* (p=3.00e-29), *CHEK2* (p=6.68e-29), *CDKN3* (p=7.78e-29), *HK2* (p=8.59e-27), *ARHGEF7* (p=1.62e-26), *KIF18A* (p=3.48e-26). We showed 28 genes with the lowest p-value through the forest map (**Figure 2A**). According to the expression levels of these 283 genes, 721 samples were clustered into different anoikis-related subtypes by consistent cluster analysis. Through the clustering heatmap and consistent cumulative distribution function (CDF) graph, we found that the CDF slope was the smallest and the clustering effect was the best when the number of clusters k was equal to 2 (**Figures 2B, C**). Through principal component analysis (PCA), we found that the clustering method could well distinguish the samples into two subtypes (**Figure 2D**) and KM survival analysis suggested a worse prognosis for ARCluster B (**Figure 2E**). These results indicated that the two anoikis-related subtypes had significant differences in the prognosis, and aroused strong interests of us in the differential expression of Args and the differential distribution of clinical information between ARCluster B and ARCluster A. We further analyzed the differences of Args between the two subtypes and found 119 differentially expressed Args. The 10 Args with the largest value of |LogFC| were *NTRK1* (LogFC=3.66), *LMO3* (LogFC=2.47), *TWIST1* (LogFC=2.46), *BIRC3* (LogFC=2.02), *HK2* (LogFC=1.94), *CCR7*(LogFC=-1.88), *ITGA8*(LogFC=-1.86), *KIF18A*(LogFC=1.82), *E2F1*(LogFC=1.78) and *UBE2C*(LogFC=1.77). 32 genes with the lowest p-value were shown by boxplot (**Figure 2F**). It could also be found that the expression levels of Args were different between ARCluster A and ARCluster B according to the heatmap (**Figure 2G**). The results also showed that ARCluster B with a poor prognosis contained more samples with age > 18 months(p<0.001), INSS stage III-IV(p<0.001), *MYCN* amplified(p<0.001) and high COG risk(p<0.001) (33, 34). However, ARCluster A with a better prognosis mainly included samples with age ≤18 months(p<0.001), INSS stage I/II/IVs(p<0.001), *MYCN* non amplified(p<0.001) and low COG risk(p<0.001)(**Figure 2G**). The above results fully indicated that children with NB could be clustered into ARCluster A with a good prognosis or ARCluster B with a poor prognosis through univariate cox regression analysis and consistent cluster analysis, which was consistent with clinical indicators such as age, COG risk, *MYCN* status and INSS stage (33, 34). These results made us interested in the study of the important pathways and key genes between the two anoikis-related subtypes.

### GSVA and GSEA analyses and immune cell infiltration in ARClusters

**Figure 3:**
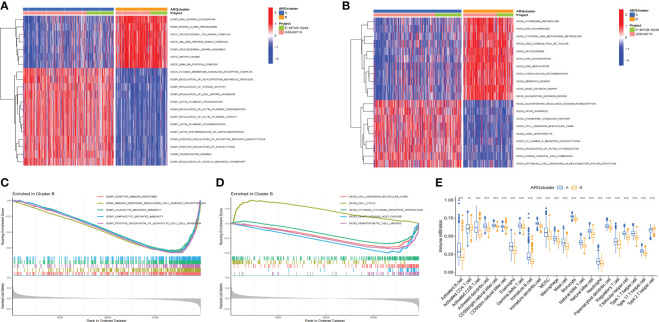
GSVA and GSEA analyses and immune cell infiltration in different ARClusters. **(A, B)**. Enrichment pathways between ARCluster B and ARCluster A through GSVA enrichment analysis. **(C, D)**. Enrichment pathways between ARCluster B and ARCluster A through GSEA enrichment analysis. **(E)**. Difference of the infiltration levels of immune cells between ARCluster A and ARCluster B through SsGSEA. **, p < 0.01; ***, p < 0.001.

In order to explore whether there were differences in the pathways between ARCluster B and ARCluster A, we performed GSVA and GSEA enrichment analyses for the two subtypes. It was found that there were obvious differences in the pathways between ARCluster B and ARCluster A through GSVA enrichment analysis. According to “c5.go.v7.4.symbols.gmt”, the five pathways with the most obvious differences were as follows: GOCC_METHYLOSOME, GOBP_ACTIN_FILAMENT_ORGANIZATION, GOBP_DNA_STRAND_ELONGATION, GOBP_REGULATION_OF_ACTIN_FILAMENT_ORGANIZATION, GOCC_SPLICEOSOMAL_TRI_SNRNP_COMPLEX (**Figure 3A**). We found that the expression level of GOBP_REGULATION_OF_CELL_MATRIX_ADHESION pathway decreased significantly in ARCluster B. According to “c2.cp.kegg.v7.4.symbols.gmt”, the most obvious five pathways differentially expressed between two subtypes were: KEGG_BASE_EXCISION_REPAIR, KEGG_SPLICEOSOME, KEGG_NUCLEOTIDE_EXCISION_REPAIR, KEGG_DNA_REPLICATION, KEGG_RNA_DEGRADATION (**Figure 3B**). We also found that the expression level of “KEGG_CELL_ADHESION_MOLECULES_CAMS” pathway related to cell adhesion was significantly reduced in ARCluster B. GSEA enrichment analysis was further performed. According to “c5.go.v7.4.symbols.gmt”, the five pathways with the most obvious expression differences were GOBP_ADAPTIVE_IMMUNE_RESPONSE, GOBP_IMMUNE_RESPONSE_REGULATING_CELL_SURFACE_RECEPTOR_SIGNALING_PATHWAY, GOBP_POSITIVE_REGULATION_OF_LEUKOCYTE_CELL_CELL_ADHESION, GOBP_LEUKOCYTE_MEDIATED_IMMUNITY, GOBP_LYMPHOCYTE_MEDIATED_IMMUNITY (**Figure 3C**). According to “c2.cp.kegg.v7.4.symbols.gmt”, the five pathways contained KEGG_HEMATOPOIETIC_CELL_LINEAGE, KEGG_GRAFT_VERSUS_HOST_DISEASE, KEGG_CELL_ADHESION_MOLECULES_CAMS, KEGG_CELL_CYCLE, KEGG_CYTOKINE_CYTOKINE_RECEPTOR_INTERACTION (**Figure 3D**). Through GSVA and GSEA enrichment analyses, it was found that there were A large number of differentially expressed pathways between ARCluster A and ARCluster B. Moreover, the results showed that the expression levels of the pathways related to cell adhesion were all reduced in ARCluster B, which was consistent with the phenomenon that malignant tumors with AR were more likely to break down the barriers of ECM and form metastasis. We analyzed the differences of the infiltration levels of immune cells between ARCluster A and ARCluster B through ssGSEA. Except for the increased level of Activated. CD4 T cell and Type2 T helper cell, the infiltration level of other immune cells in ARCluster B decreased significantly (**Figure 3E**). These results fully showed that there were obvious differences in the infiltration of immune cells between different anoikis-related subtypes of NB, and the changes of TME might play an important role in breaking down the barriers of ECM and forming AR.

### Hub genes in anoikis-related subtypes

**Figure 4:**
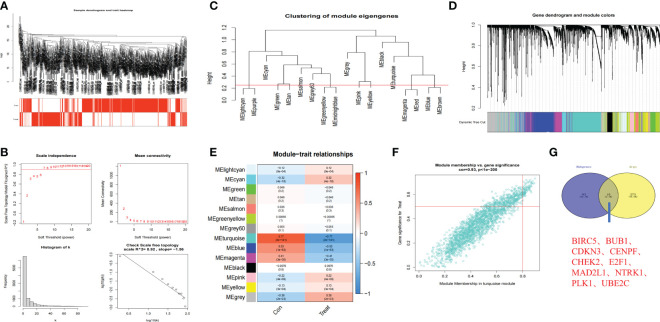
Hub genes in anoikis-related subtypes based on WGCNA. **(A)** Clustering dendrogram of 721 samples. **(B)** Construction of scale-free network (Soft threshold value=7, R^2 =^ 0.92). **(C, D)**. 18 anoikis-related modules of NB were screened and further combined into 14 modules (Red line in C: MEDissThres = 0.25). **(E)** Relationships between anoikis and 14 modules. Turquoise module was most closely related to anoikis in NB. **(F)** Scatterplot analysis of Turquoise module. Key genes were filtered in the upper right region of GS > 0.5 and MM > 0.8. **(G)** The Venn plot of 10 hub genes in Turquoise module and 283 Args.

We further screened the hub genes that might play important roles in anoikis-related subtypes through WGCNA. The remaining samples were clustered after removing outliers (**Figure 4A**). When the threshold value was 7, R^2 =^ 0.92, the gene expression was the most consistent with the scale-free network (35) (**Figure 4B**). Through the construction of scale-free network, we obtained a total of 18 modules closely related to anoikis and further combined the 18 modules into 14 modules according to the standard of Height=0.25 (**Figures 4C, D**). Among the 14 modules, MElightcyan, MEcyan, MEpink, MEyellow, and MEgrey were positively related to anoikis, while MEturquoise, MEblue, and MEmagenta were negatively related to anoikis. Among all modules, MEturquoise module had the largest number of genes and was the most closely related to anoikis (correlation index=-0.77) (**Figure 4E**). According to the screening criteria of GS>0,5 and |MM|>0.8, 93 core genes were screened from the MEturquoise module (**Figure 4F**). We further crossed the prognosis-related Args with the 93 core genes and finally obtained 10 anoikis-related hub genes (**Figure 4G**): *BIRC5* (GS=0.62, MM=-0.81), *BUB1* (GS=0.60, MM=-0.80), *CDKN3* (GS=0.64, MM=-0.82), *CENPF* (GS=0.61, MM=-0.82), *CHEK2* (GS=0.66, MM=-0.85), *E2F1* (GS=0.64, MM=-0.83), *MAD2L1* (GS=0.64, MM=-0.81), *NTRK1* (GS=-0.67, MM=0.82), *PLK1* (GS=0.65, MM=-0.85) and *UBE2C* (GS=0.60, MM=-0.81).The above results indicated that there were multiple modules affecting anoikis between two anoikis-related subtypes of NB, and the MEturquoise module had the most obvious inhibitory effect on the anoikis of NB. The screening of the hub genes in this module would play an important role in our subsequent research on the mechanism of AR in NB.

### Construction of an accurate anoikis-related model

**Figure 5:**
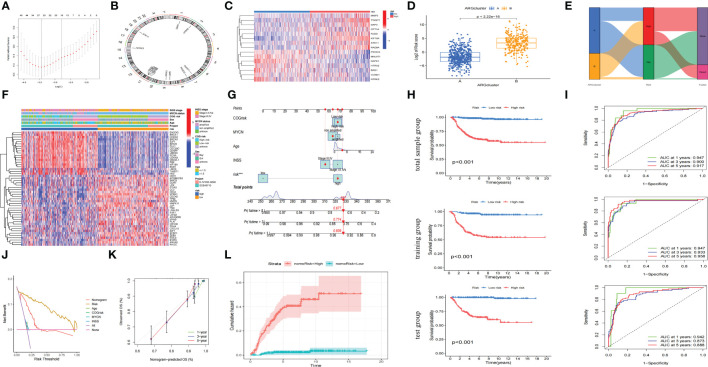
Construction of an accurate anoikis-related model. **(A)** 26 key genes screened through further cross-validation (alpha=1). **(B)** The locations of 15 genes in chromosomes obtained by multivariate cox regression analysis. **(C)** Heatmap of the distribution of the 15 genes involved in model construction between the high riskscore and low riskscore groups. **(D)** Boxplot of riskscore distribution of samples in different ARClusters. **(E)** Alluvial diagram of subtypes and living status. **(F)** Heatmap of clinical information and differentially expressed Args between two risk subgroups. **(G)** Nomogram of the commonly used clinical indicators and the risk level calculated by anoikis-related model. **(H)** OS analysis of two risk subgroups in the total sample group, training group and test group. **(I)** The time-dependent ROC curves for OS at 1-, 3-, and 5-years in the total sample group, training group and test group. **(J)** Net benefit (y-axis) as calculated are plotted against the threshold probabilities of patients having 5-year survival on the x-axis. **(K)** Calibration curve of OS at 1-, 3-, and 5-years for the anoikis-related model in the total sample group. **(L)**. Cumulative hazard curve represented the probability of survival risk with the prolongation of survival time. ***, p < 0.001.

We had found that there were two anoikis-related subtypes with different prognoses in NB through the above cluster analysis. Therefore, we hoped to construct a prognostic model that could classify NB into anoikis-related subtypes with different prognoses through their transcriptome data. Firstly, we randomly divided 721 cases of NB into the training group and the test group with the same sample size, and constructed lasso regression analysis based on the expression levels of 283 Args and the prognoses of these children. We got a total of 26 key genes through further cross-validation (alpha=1). The multivariate cox regression analysis was carried out between the expression of these 26 key genes and the prognoses of these patients. Finally, we obtained 15 key Args for building the model and listed the location of each gene (**Figures 5A, B**): *STK11*, *CCND1*, *PIK3CG*, *DAPK1*, *FADD*, *CPT1A*, *TWIST1*, *BAG1*, *RAD9A*, *MALAT1*, *NTRK3*, *MMP3*, *HTRA2*, *KIF18A*, *DAP3*. The weight of each gene in this model was stated in **Table 1**. By calculating the riskscore of each sample, we divided the samples of training group and test group into high-risk group and low-risk group according to the median value of each group. The results suggested that the expression levels of 15 Args participating in the construction of this model were different between high and low-risk groups (**Figure 5C**), and the riskscores in ARCluster B were significantly higher than those in ARCluster A (**Figure 5D**). Almost all of the samples in ARCluster B were classified into the high-risk group, and most of the samples in ARCluster A were classified into the low-risk group. Almost all the dead patients came from the high-risk group, while the majority of the surviving children belonged to the low-risk group (**Figure 5E**). We found that Args differentially expressed in ARCluster A and ARCluster B groups also had consistent differences between high-risk and low-risk groups, and the clinical indicators related to poor prognosis including ≥ 18 months, *MYCN* amplified, high COG risk, INSS stage III-IV were mainly enriched in the anoikis-related high-risk group (**Figure 5F**), which was consistent with our previous conclusions. According to the commonly used clinical indicators: Age, *MYCN* status, COG risk, INSS stage and the risk level calculated by anoikis-related model, we drew the nomogram to comprehensively evaluate the prognoses of children with NB. Different clinical indicators were given different points in the nomogram. The probabilities of the 1-, 3-, and 5-year survival were predicted by the accumulated scores. It was found that the scores of the two opposite states of other indicators were very close except for the risk level. The nomogram showed that the risk level was an independent diagnostic factor and synergistically predicted the survival probability of NB patients. (**Figure 5G**). KM survival analysis indicated that the prognoses of patients with high riskscores were poor (p<0.001 in three groups) whether in the total sample group, training group or test group (**Figure 5H**). AUC values at 1-year, 3-years, 5-years in the total sample group (0.947, 0.900, 0.917), training group (0.947, 0.933, 0.958), and test group (0.942, 0.873, 0.888) indicated that the model we built could well predict the prognoses of children with NB (**Figure 5I**). By drawing the decision curve (**Figure 5J**) and calibration curve (**Figure 5K**), we could also find that the anoikis-related model we built could well predict the 1,3,5-year survival rate of children with NB. The cumulative hazard curve showed that the overall survival (OS) risk of children in high-risk group increased gradually with the prolongation of survival time (**Figure 5L**). The above results suggested that this anoikis-related model could accurately reflect the prognoses of children with NB, which laid the foundation for the popularization of this model and the researches on the potential mechanisms and immune microenvironment of NB in high-risk group.

**Table 1:** 15 key genes involved in anoikis-related prognostic model construction.

GENE ID	Official full name	Ensemble id	Weight
STK11	serine/threonine kinase 11	ENSG00000118046	0.994623511563205
CCND1	cyclin D1	ENSG00000110092	-0.537885548425383
PIK3CG	phosphatidylinositol-4,5-bisphosphate 3-kinase, catalytic subunit gamma	ENSG00000105851	-0.369580805368059
DAPK1	death-associated protein kinase 1	ENSG00000196730	-0.546016481616927
FADD	Fas (TNFRSF6)-associated via death domain	ENSG00000168040	0.650215657607045
CPT1A	carnitine palmitoyltransferase 1A (liver)	ENSG00000110090	0.488264733173891
TWIST1	twist family bHLH transcription factor 1	ENSG00000122691	0.141516754247007
BAG1	BCL2-associated athanogene	ENSG00000107262	-1.56407856752709
RAD9A	RAD9 homolog A (S. pombe)	ENSG00000172613	0.919230796386681
MALAT1	metastasis associated lung adenocarcinoma transcript 1	ENSG00000251562	-0.845810455874506
NTRK3	neurotrophic tyrosine kinase, receptor, type 3	ENSG00000140538	-0.27217957462717
MMP3	matrix metallopeptidase 3 (stromelysin 1, progelatinase)	ENSG00000149968	0.187574893822123
HTRA2	HtrA serine peptidase 2	ENSG00000115317	-1.63688276274086
KIF18A	kinesin family member 18A	ENSG00000121621	0.670454648286514
DAP3	death associated protein 3	ENSG00000132676	0.836468483318045

### Validation of the anoikis-related model

**Figure 6:**
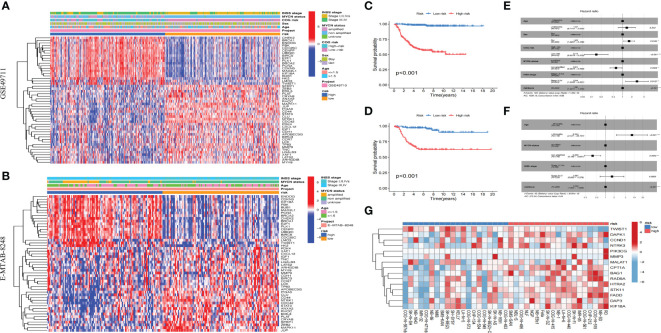
Validation of the anoikis-related model. **(A, B)**. Heatmap of clinical information and differentially expressed Args between two risk subgroups in GSE49710 and E-MTAB-8248. **(C, D)**: KM survival analysis of two risk subgroups in GSE49710 and E-MTAB-8248. **(E, F)**: Forest plots of multivariable cox regression analyses of the clinical features and riskscores calculated by the anoikis-related model in GSE49710 and E-MTAB-8248. **(G)**: Riskscores of 39 NB cell lines in GSE89413 cohort according to the constructed model. *, p < 0.05; **, p < 0.01; ***, p < 0.001.

We found that the model constructed in this study could effectively divide the samples of GSE49710 and E-MTAB-8248 cohorts into high-risk group and low-risk group (**Figures 6A, B**), and the prognoses of children in high-risk group were worse than that in low-risk group (**Figures 6C, D**). Args differentially expressed between ARCluster A and ARCluster B were also differentially expressed between high and low-risk groups of the GSE49710 and E-MTAB-8248 cohorts according to the heatmap (**Figures 6A, B**). Clinical indicators closely related to poor prognoses of children: ≥18 months, *MYCN* amplified, high COG risk, INSS stage III-IV were also enriched in high-risk group of both cohorts (**Figures 6A, B**). The results of independent prognostic analysis suggested that sex, *MYCN* status, COG risk, INSS stage and anoikis-related riskscore could be used as independent prognostic indicators in the GSE49710 cohort (**Figure 6E**). Age, *MYCN* status and anoikis-related riskscore were independent prognostic indicators in the E-MTAB-8248 cohort (**Figure 6F**). 39 NB cell lines in GSE89413 cohort were also scored according to the constructed model. RD, NB-SD, COG-N-519, CHP-212, COG-N-561, IMR-05, SK-N-FI, COG-N-440, LA-N-6, RD, NB-SD, COG-N-519, CHP-212, COG-N-561, IMR-05, SK-N-Fi, COG-N-440, LA-N-6 and Nb-1 were the top ten NB cell lines with the highest riskscore, while COG-N-557nb, SK-N-SH, NB-1643, IMR-32, COG-N-471nb, NB-69, NMB, SMS-KAN, SK-N-BE (2) and SH-SY5Y were the top ten cell lines with the lowest riskscore (**Figure 6G**). The above results fully indicated that the anoikis-related model constructed by us could generally predict the prognoses of children with NB, and the scores obtained by this model could be used as an independent indicator to better predict the prognosis. The above results also suggested that there were generally two anoikis-related subtypes in the population and cell lines of NB, among which the subtype with high riskscore and poor prognosis was likely to be closely related to the AR. The classification of NB cell lines would help us study the mechanism of AR in NB.

### Correlation between immune cell status and different riskscores

**Figure 7:**
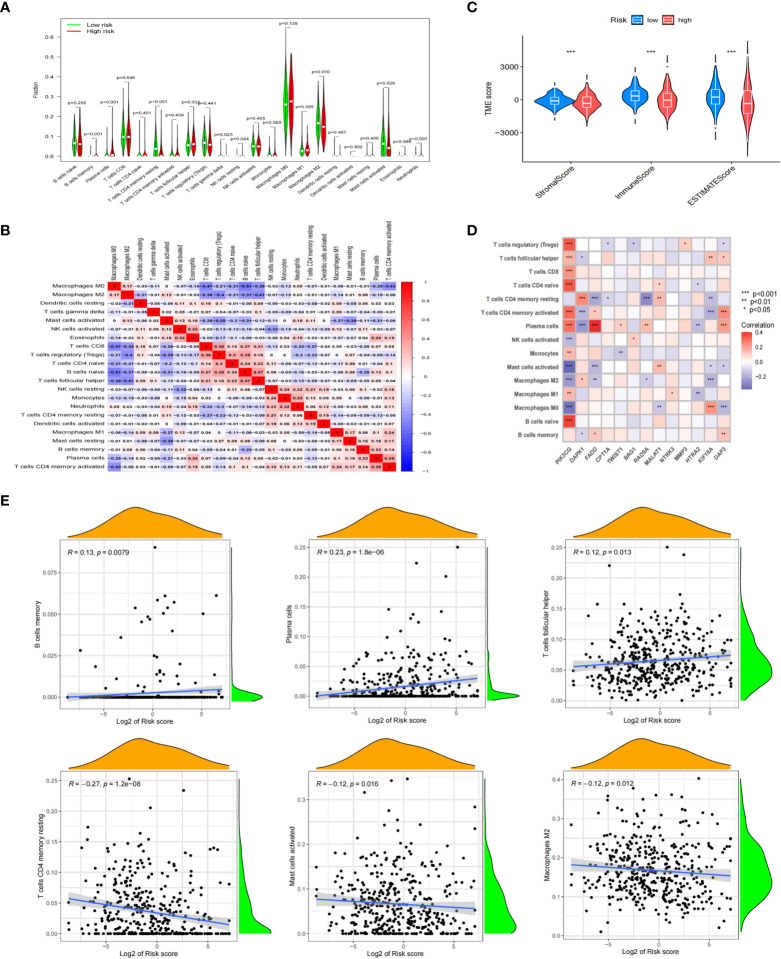
Correlation between immune cell status and different riskscores. **(A)** Analyzing the immune cell infiltration levels of NB samples from different anoikis-related risk groups by CIBERSORT algorithm. **(B)** Correlations between immune cells. **(C)** Differences of TME between different risk groups. **(D)** Analyzing the correlations between 15 Args participating in the model construction and immune cells. **(E)** Relationships between 6 kinds of immune cells and the riskscores of patients. *, p < 0.05; **, p < 0.01; ***, p < 0.001.

Previous studies had shown that TME was closely related to the metastasis of tumor cells, and AR was an important mechanism for metastasis. Therefore, understanding the TME characteristics of anoikis-related subtypes of NB was very important to immunotherapy for anoikis-related subtype of NB with a poor prognosis. CIBERSORT algorithm was used to analyze the immune cell infiltration level of NB samples from different anoikis-related risk groups. The results showed that B cells memory, Plasma cells, T cells follicular helper, NK cells resting, and Neutrophils were highly expressed, while T cells CD4 memory resting, T cells gamma delta, Macrophages M2 and Mast cells activated were weekly expressed in anoikis-related subtype with high riskscore (**Figure 7A**). We also found that there were correlations between immune cells, especially positive relationship between B cells naive and T cells follicular helper (correlation index=0.37). But the negative relationship between B cells naive and Macrophages M0 was more obvious (correlation index=-0.51) (**Figure 7B**). The estimate algorithm was used to further analyze the differences of TME between different risk groups. The results showed that the estimate score of high-risk group was lower, and the content of stromal cells and immune cells in tumor tissue was lower than that of low-risk group (**Figure 7C**). By analyzing the correlations between 15 Args participating in the model construction and immune cells, we found that except *STK11* and *CCND1*, the other 13 Args were all related to the infiltration levels of immune cells. In particular, the expression level of *PIK3CG* was positively correlated with the infiltration levels of T cells regulatory (Tregs), T cells follicular helper, T cells CD8, T cells CD4 naive, T cells CD4 memory activated, Plasma cells, Monocells, Macrophages M1, B cells naive, and negatively correlated with the expression levels of NK cells activated, Mast cells activated, Macrophages M2, Macrophages M0 (**Figure 7D**). By further analyzing the correlation between the infiltration levels of 23 kinds of immune cells and the riskscores of patients, we found that a total of 6 kinds of immune cells were related to the riskscore. B cells memory (R=0.13, p=0.0079), Plasma cells (R=0.23, p=1.8E-6) and T cells follicular helper (R=0.12, p=0.0013) were positively correlated with the riskscore, while T cells CD4 memory resting (R=-0.27, p=1.2E-8), Macrophages M2 (R=-0.12, p=0.0012) and Mast cells activated (R=-0.12, p=0.0016) were negatively correlated with the riskscore (**Figure 7E**). The above results fully demonstrated that TME and immune cell infiltration level were different between anoikis-related subtypes. The key genes involved in the construction of anoikis-related model were also related to the infiltration levels of immune cells. The differences of the TME and immune cell infiltration level might be important factors leading to the AR of NB, and the key genes for constructing the model might play important roles in the above mechanisms.

### Chemotherapeutic response analysis

**Figure 8:**
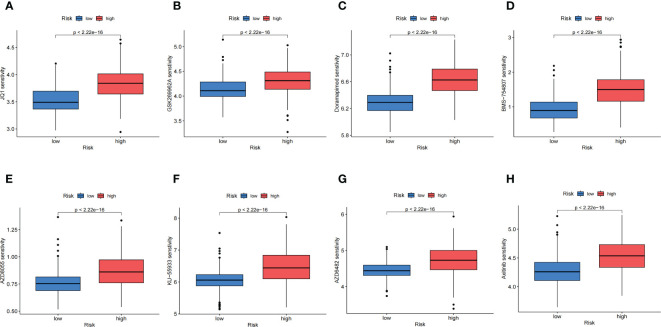
Chemotherapeutic Response Analysis. **(A-H)**. The eight predicted chemotherapeutic drugs with the highest drug sensitivity in the high-risk group.

According to the results calculated based on the GDSC database, we predicted that the anoikis-related high-risk group and low-risk group had different drug sensitivities to 158 kinds of chemotherapeutic drugs. The low-risk group had higher sensitivity to most of the predicted drugs, while the high-risk group had higher sensitivity to only17 kinds of drugs. The 8 kinds of chemotherapeutic drugs with the highest drug sensitivity in high-risk group were: JQ1, GSK269962A, Doramapimod, BMS-754807, AZD8055, KU-55933, AZD6482 and Axitinib (**Figure 8**). The above results indicated that these selected chemotherapeutic drugs might have better therapeutic effects on NB in high-risk group, and might have potential targeted therapeutic effects on the AR of NB.

## Discussion

NB is the most common solid extra-cranial malignancy in infants (36). At present, there are many treatments for NB, including surgery (4, 5), chemotherapy (7, 8), bone marrow transplantation (10), immunotherapy (11–16) and so on. However, the five-year survival rate is still low even with multiple therapies for high-risk NB, especially those with metastasis. Therefore, exploring the mechanisms of metastasis has a high clinical significance for the treatment of NB. In recent years, as an important mechanism of metastasis, AR has become a hot topic of scientific research and has been paid more and more attentions by scholars. Anoikis can prevent normal cells and tumor cells from leaving the ECM, suppress their distant colonization and growth, while malignant tumor cells colonizing in organs other than the primary site can get rid of the regulation of anoikis. In recent years, the AR has been found in many solid tumors, including breast cancer (37, 38), prostate cancer (39, 40), gastric cancer (41, 42), liver cancer (43, 44), renal cancer (45, 46) and so on. Some scholars have also found this phenomenon in NB (31, 32), but there is a lack of research on the relevant mechanism. In this study, transcriptome microarray data of children with NB were used to construct an anoikis-related prognostic model for the first time. Studies had shown that the model constructed by us could well predict the prognoses of children and provide an auxiliary role for the study of mechanisms related to the AR.

This study followed the principle of large sample size and complete clinical information when we selected the cohorts, and finally included 721 cases for consistency cluster analysis. The Args obtained were also strictly screened, and the genes less related to anoikis were removed, which ensured that the constructed model had high accuracy and high correlation with anoikis. The results of consistent cluster analysis showed that there were two anoikis-related subtypes with completely opposite prognosis in the population of NB. Through the analysis of the distribution of clinical characteristics of different subtypes, we found that the clinical information indicating a poor prognosis was enriched in the poor prognostic subtype, and the distribution of Args in the two subtypes was also significantly different. By dividing samples into these two subtypes, we could more effectively and accurately analyze the changes of core genes, key pathways and immune microenvironment related to the anoikis of NB. But a small number of children with poor prognostic indicators were enrolled in the good prognostic subtype. We thought that the expressions of Args could distinguish between more and less aggressive MYCN or stage III-IV tumors, or maybe a certain subgroup of aggressive tumors could be predicted based on Args.

Through the enrichment analysis of GSVA and GSEA for the two subtypes, we found that there were significant differences in the expression levels of pathways between the two subtypes, and the expression level was significantly reduced especially in the pathway of cell adhesion, which was consistent with the phenomenon of AR. The analysis of these differentially expressed pathways will help us better understand the important roles of AR in the metastasis of NB. By analyzing the immune cell infiltration of the two subtypes, we speculated that the occurrence of anoikis might be closely related to the TME of NB, especially the changes of immune cell infiltration. We also found that there were multiple modules that played important roles in anoikis in the subtype with a poor prognosis through WGCNA analysis. Ten hub Args that played important roles in the mechanism of anoikis in NB were obtained. Anoikis is actually a kind of responses of cells to their loss of contact by different signal pathways, and cell cycle regulation is also an important way for cells to generate anoikis or AR (47, 48). Previous studies have mentioned that *BIRC5*, *BUB1*, *CDKN3*, *CENPF*, *PLK1*, *CHEK2*, *Ntrk1*, *E2F1* and *UBE2C* are closely related to cell cycle. However, the functions of these genes are not single and these genes may play other functions such as promting anoikis or AR in additon to regulating cell cycle. For example, Survivin (BIRC5) is required for enhancing AR in ovarian cancer cells and hepatocellular carcinoma cells (24, 49). CHEK2 is a mediator of anoikis of intestinal epithelial cells and is associated with the progression of papillary thyroid cancer by promoting anoikis (50, 51). The protein encoded by *E2F1* which plays a crucial role in the control of cell cycle is a member of the E2F family of transcription factors. However, current studies have also found that E2F1 is closely related to anoikis (52), and CDKN3, as the upstream of E2F1, regulates the expression of E2F1 (53). The PI3K/Akt signal pathway is one of the most important downstream targets of TrkA translated by *Ntrk1 (* 54, 55), and mTOR which is thought to be directly involved in anoikis is the downstream target of PI3K/Akt pathway (56, 57). Studies have shown that PLK1 is closely related to cell mitosis, but recent studies have also found that PLK1 protects esophageal cancer cells from anoikis through regulating β-Catenin protein levels by inhibiting their degradation (58). BUB1 can form polymer with PLK1 and promote the function of PLK1 (59). Recent studies have also found that UBE2C/ZEB1/2 signal axis plays an important role in the metastasis and AR in cervical cancer (60). Silencing of CENPF could simultaneously improve sensitivity to anoikis-induced apoptosis in human PC3 cells (61). All these results indicated that the hub genes involved in this study were indeed closely related to anoikis.The study of these genes will help us better understand the role of hub genes in the mechanisms of anoikis in NB.

A large number of previous studies have shown that TME not only plays an important role in the metastasis of NB (62, 63), but also is closely related to the drug resistance (64–66), especially immunotherapy drugs (65, 66). Therefore, the study of TME in the anoikis-related subtypes will help us better understand the mechanism of AR in NB and guide the screening of targeted therapeutic drugs. Through the analysis of TME, we found that the infiltration levels of some immune cells in the two groups were significantly different. The infiltration levels of some immune cells were also correlated with the riskscore of each sample, and 13 of the 15 genes involved in the model construction were correlated with the infiltration levels of some immune cells. These findings with statistical differences will provide guidance for us to further analyze the correlation between TME and AR of NB.

At present, we still face many challenges for the clinical treatments of NB, especially for the refractory and relapsed high-risk NB (67, 68), which is also the focus of scientific research. In this study, we found that there was a subtype of NB with a poor prognosis, which was closely related to anoikis. If there are effective chemotherapeutic drugs or targeted therapeutic drugs, the prognosis will be greatly improved for these children. Through drug sensitivity prediction, we found that most of the drugs with statistical significance were sensitive to the subtype of NB with good prognosis, while only a small number of drugs were sensitive to the poor prognosis subtype of NB. At present, there is no reseach on directly applying any of the eight drugs obtained to the study of anoikis. However, the targets of some drugs may be related to anoikis. For example, the PI3K/Akt/mTOR signaling pathway promotes the development of AR (57). Current studies have found that AZD6482 is a selective inhibitor of PI3K (69, 70). Recent studies have shown that AZD8055, as a potent dual mTORC1-mTORC2 inhibitor (71), can inhibit NB proliferation *in vitro* and *in vivo* by targeting mTOR (72). Overexpression of mTOR in non-transformed wt fibroblasts promoted AR, and mTOR knockdown in MEFs deficient in all retinoblastoma-family members restored anoikis (73). GSK269962A is a selective inhibitor of ROCK (74), and multiple studies have shown that ROCK is closely related to AR (75, 76). Doramapimod is a selective inhibitor of P58 MAPK (77) and ERK/MAPK signaling was found to operate downstream of ErbB2 and EGFR to protect cells from anoikis (78). Some studies have shown that IGF-1R is associated with AR (79, 80), and BMS-754807 is an important selective inhibitor of IGF-1R (81). Recent study has also found that foretinib, as an orally available multikinase inhibitor of c-Met and VEGFR-2, blocks proliferation, induces anoikis and impairs ovarian cancer metastasis (82). Axitinib, as a selective inhibitor of VEGFR, may inhibit AR (83). In the later stage, further experiments *in vivo* and *in vitro* on these drugs may help us better understand the relevant mechanisms of AR in NB and guide the selection of chemotherapeutic drugs and molecular targeted drugs for the anoikis-related NB subtype with poor prognosis.

## Conclusion

In conclusion, this study clustered NB patients into two anoikis-related subtypes with different prognoses based on consistency clustering analysis, and analyzed the differences in clinical information, hub genes, signal pathways and immune microenvironment between the two subtypes. Subsequently, we constructed an anoikis-related model that could evaluate the prognoses of children through 15 Args, further verified the accuracy, popularization and application of this model, analyzed the differences of TME between two risk groups, and screened 17 drugs with high drug sensitivity in high-risk group. The model constructed in this study has a good guiding significance for the risk staging and prognosis evaluation of NB, a high auxiliary role in the study of the mechanism related to the AR, and a good reference significance for the selection of chemotherapeutic and targeted therapeutic drugs for NB.

## Data availability statement

The datasets presented in this study can be found in online repositories. The names of the repository/repositories and accession number(s) can be found in the article/**Supplementary Material**.

## Author contributions

LH, BJ and YF: conceptualization, methodology, writing-reviewing and editing. JC, MS, CC and MK: investigation, data curation, writing-original draft preparation. JC, CC, BQ, JS, XM and JZ: visualization, validation, supervision, software. All authors contributed to the article and approved the submitted version.
